# Scrub typhus in Nan province (Thailand): Seventeen years of data to understand the impact of land cover change

**DOI:** 10.1371/journal.pntd.0013552

**Published:** 2025-09-18

**Authors:** Nolwenn Blache, Karine Chalvet-Monfray, Rawadee Kumlert, Soawapak Hinjoy, Serge Morand

**Affiliations:** 1 Université Clermont Auvergne, UMR Territoires, VetAgro Sup, Lempdes, France; 2 Université de Lyon, UMR EPIA, INRAE, VetAgro Sup, Marcy l’Etoile, France; 3 Division of Vector-Borne Diseases, Department of Disease Control, Ministry of Public Health, Nonthaburi, Thailand; 4 Department of Disease Control, Ministry of Public Health, Nonthaburi, Thailand; 5 IRL2021 HealthDEEP, CNRS – Kasetsart University – Mahidol University, Bangkok, Thailand; 6 Faculty of Veterinary Medicine, Kasetsart University, Bangkok, Thailand; 7 Faculty of Tropical Medicine, Mahidol University, Bangkok, Thailand; Tufts Medical Center, UNITED STATES OF AMERICA

## Abstract

**Background:**

Scrub typhus, caused by *Orientia tsutsugamushi* and transmitted by chigger mites (*Leptotrombidium*), is a major health problem in northern Thailand, particularly in Nan province. Land cover change, by altering the ecosystem, could affect the ecology of the vector and consequently the risk of scrub typhus transmission.

**Methodology/principal findings:**

This study investigated the impact of land cover changes on scrub typhus transmission in 2.5 km buffer zones around each village of Nan Province between 2003 and 2019. Using the open land cover data of the European Spatial Agency Climate Change Initiative (ESA CCI), we quantified land cover composition and land cover changes and integrated public health data on scrub typhus cases, as well as information on elevation, population, and slope. Generalized Additive Models were applied to assess the effects of land cover changes on annual scrub typhus cases. Scrub typhus cases increased significantly during the study period, peaking in 2012 and 2016, mainly in mountainous areas rather than in the Nan River valley. Land cover associated with cases included shrubland, mosaic land, broadleaf forest, and needleleaf forest. Cases increased with shrubland and mosaic land, displayed an inverted U-shaped relationship with broadleaf forest, and decreased with needleleaf forest. Key land cover change factors included shrubland transitions, population, and geographic interactions. Reforestation (from shrubland to broadleaf forest) showed an inverted U-shaped relationship with cases, whereas stable broadleaf forest and loss of shrubland to grassland became non-significant. Male population increased cases.

**Conclusions/significance:**

This study highlights the importance of land cover changes in understanding disease transmission and suggests that landscapes disturbance may create optimal conditions for *O. tsutsugamushi* transmission. This is a novel regional-scale exploration of land cover impacts on scrub typhus in Thailand.

## Author summary

Scrub typhus, caused by the bacterium *Orientia tsutsugamushi*, is a vector-borne disease transmitted to humans exclusively by chigger mites. Despite one million annual cases, it remains a neglected tropical disease due to limited knowledge of vector ecology and transmission risk factors. Thailand is particularly affected by this disease where the pathogen actively circulates mainly in the northern region known as a rural endemic area. Focusing on Nan province, part of the endemic region, we investigated between 2003 and 2019, how the composition of land cover and land cover changes in the surroundings of the villages impact the number of scrub typhus human cases. Using satellite-derived land cover data and public health records, we found that important proportion of shrubland and mosaic land as well as an intermediate proportion of broadleaf forest were at risk for human health. When further investigating the role of land cover change, we discovered that areas with moderate reforestation from shrubland to forest increased scrub typhus cases, whereas stable forest cover from 2003–2019 showed no significant effect. These findings highlight the critical role of land cover and land cover changes in shaping disease risk, providing new insights into the ecological drivers of scrub typhus outbreaks.

## Introduction

Scrub typhus is a vector-borne disease transmitted to humans by chigger mites of the genus *Leptotrombidium*. The pathogen responsible for scrub typhus is a strictly intracellular bacteria called *Orientia tsutsugamushi* from the family of Rickettsiae*.* It is during the feeding process that the 6-leg larval stage vector also known as “chiggers”, transmits the pathogen to humans. The disease manifests itself with flu-like symptoms, a skin eschar and death in 6% of the cases in the absence of antibiotic treatment [1]. A commonly cited estimate records one million new cases per year worldwide and potentially one billion people at threat [2]. The pressure is the highest in the endemic are known as the Tsutsugamushi Triangle encompassing Japan, Southeast Asia and Australia. Thailand is particularly impacted by the disease, with the Ministry of Public Health recording 103,345 cases between 2003 and 2017 [3] and up to 77% of the villagers estimated to have been exposed to the pathogen in the worst-affected regions [4]. Half of the total scrub typhus cases reported between 2003 and 2018 occurred in the northern region. This area also included the five provinces with the highest annual cases number, making it an significant endemic area of Thailand [3].

Human cases of scrub typhus have been correlated with vector abundance in several studies [5]. Species richness of chigger mites and their infection rates with *Orientia tsutsugamushi* have also been identified as risk factors [6,7]. Chiggers can become infected by feeding on an infected host [8]. However, laboratory studies have suggested that they are primarily infected by transstadial and transovarial transmission, allowing the infection to be maintained over multiple generations [9]. *Leptotrombidium* mites spent 99% of their time living free in the environment and 1% feeding on vertebrate hosts. When free-living, chiggers require stable and suitable temperature and humidity conditions for their survival. They also need to encounter a host on which they can feed to complete their cycle. Early local studies in several regions of Southeast Asia identified chigger habitats as forested and shrubland areas, fallow land, neglected gardens or plantations, flooded meadows, hedgerows, marginal habitats and ecotones [10–12]. Secondary vegetation growth resulting from land cover change and favouring chiggers has also been identified as a risk factor in local studies [13]. Vectors may then benefit from the forest and associated tree cover because of the microclimate they provide and the small mammals and birds they support, making them a risky habitat if humans are exposed [14]. Few studies have investigated the impact of land cover and land cover change on scrub typhus at the regional scales, such as in China [15,16], Taiwan [17], South Korea [18] or Thailand [3]. However, to our knowledge, no studies have been conducted at local or village level which could be relevant given the low mobility of chiggers and the high heterogeneity of human cases within regions.

This study aimed to quantify the role of land cover and land cover change on scrub typhus cases in an endemic area of North Thailand. Instead of aggregating the scrub typhus cases at the subdistrict level, we used a 2500m radius buffer zone around each village of Nan Province that could encompass areas where villagers are mainly exposed during their agricultural activities. Based on previous literature, we hypothesized that 1) forest-related land covers have a higher burden of scrub typhus; 2) fast changing land covers such as secondary growth vegetation or fragmented landscapes increase the burden of scrub typhus compared with more stable land covers. Land cover and land cover transitions between 2003 and 2019 were quantified within these buffer zones. We combined passive public health surveillance data of scrub typhus cases with land cover, land cover transitions, and topographic data in two spatiotemporal models: one assessing the impact of land cover considering years and the other examining the effects of land cover transitions.

## Methods

### Study area

**Fig 1:**
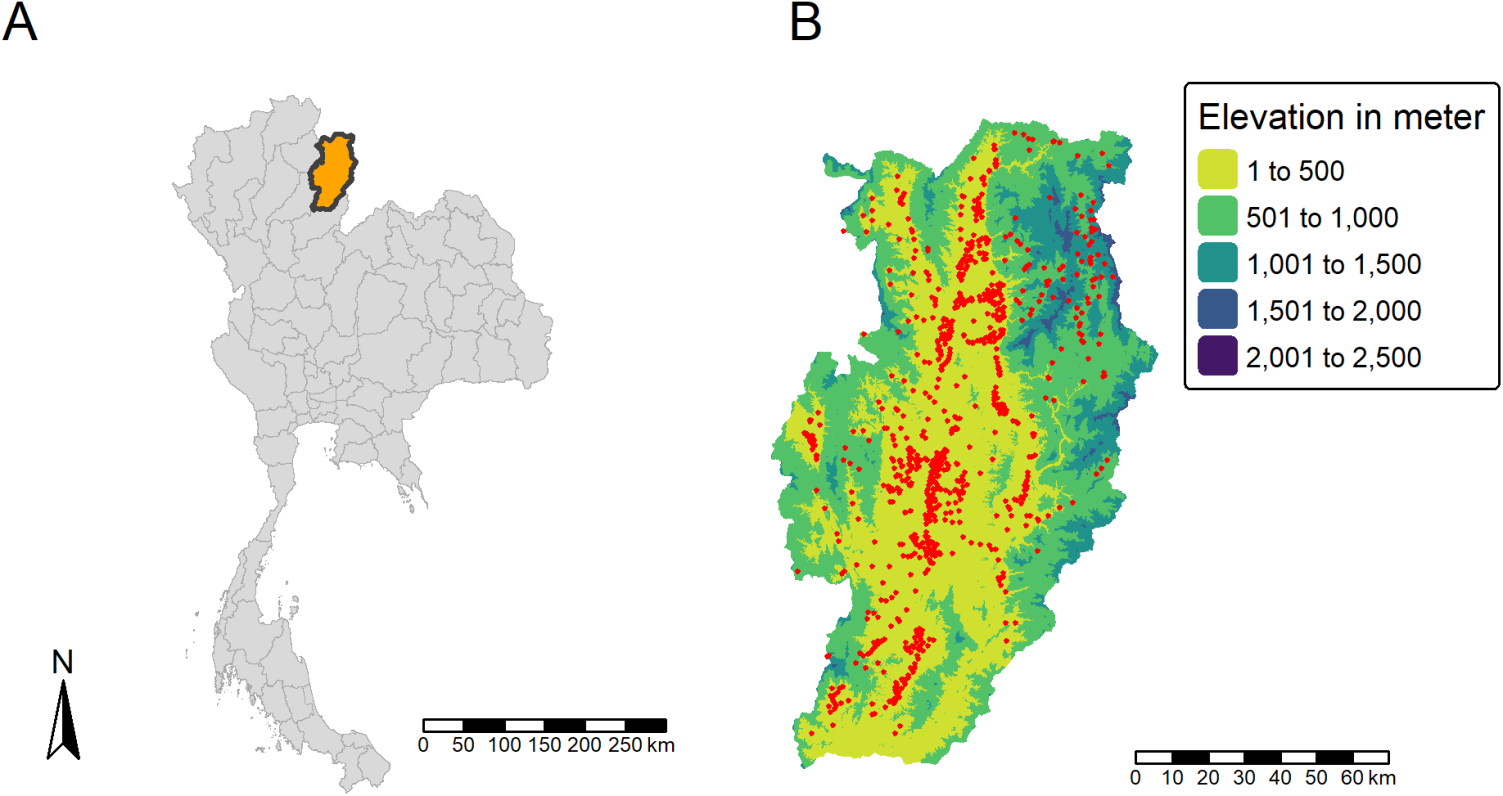
Maps of (A) Thailand with Nan province highlighted in orange and (B) Nan province elevation and villages (n = 905). The red dots represent the villages positions. The base layer of the map is available at https://data.humdata.org/dataset/cod-ab-tha.

Nan Province (19.15° N, 100.83° E) is located in the northern part of Thailand (Fig 1A), bordered by Phayao and Phrae Provinces to the West, Uttaradit Province to the South and Lao PDR to the Northeast. Nan Province covers approximately 12,100 km^2^, with 15 districts, 99 sub-districts, and 905 villages. The population reached 478,227 inhabitants in 2019 [19] and most of the employed people work in agriculture [20,21]. The Nan River flows through the province from north to south, creating a flat agrarian valley surrounded by mountainous and foothill areas (Fig 1B). The average temperature is 24.8°C with a minimum temperature of 16.4°C (January) and a maximum temperature of 33.8°C (recorded in April) for the period 1991–2021. The average annual rainfall for the same 30-year period was 1,490 mm, with most of the falling during the wet season from May to September. Nan Province is recognized as being severely affected by scrub typhus. In 2019, Nan Province had the second highest reported cases per 100,000 inhabitants of Thailand, reaching 114.71 [22].

### Data collection

We retrieved the number of scrub typhus human cases between 2003 and 2019 from the National Disease Surveillance System (Division of Epidemiology, Department of Disease Control, Ministry of Public Health of Thailand) [23]. The number of human scrub typhus cases was aggregated annually for each village in the province. The cumulated cases per village between 2003 and 2019 was also calculated. Human cases included both confirmed and probable cases as defined by the ICD-10: A75.3 and explained in Wangrangsimakul et al. [3]. The location (longitude, latitude) of each village of the province as well as the population per village were retrieved from national census data. We estimated the elevation in meters of each village and the mean slope in degrees for each village buffer from the Environment Operations Center (www.gms-eoc.org) based on Version 4.1 of NASA’s Shuttle Radar Topographic Mission (SRTM) elevation dataset. We used the land use land cover time series maps provided by the European Space Agency Climate Change Initiative (ESA CCI) to extract land cover data [24]. These global land cover maps were built at a spatial resolution of 300m on an annual basis between 1992–2020. We downloaded the 2003–2019 raster maps to match the temporal range of scrub typhus cases. The maps initially encompass 37 global or regional land cover classes numbered from 0 to 220 shown in Supporting Information S1 Fig. We selected and aggregated the following nine land cover classes: Forest broadleaf (corresponding to the n°50, 60, 61 and 62 in the ESA CCI map products classification); Forest needleleaf (n°70, 71, 72, 80, 81 and 82); Cropland rainfed (n°10); Cropland irrigated (n°20); Shrubland (n°120, 121, 122, 152 and 180); Grassland (n°153 and 120); Mosaic (n°30, 40, 100, 110, 150 and 151); Urban areas (n°190) and Water bodies (n°210). For each village and for each year, the area of the nine new land cover classes was extracted in the 2500 m buffer using R software [25] and the *sf* package v1.0-15 [26] and *terra* packages v1.7-55 [27]. By comparing raster maps from 2003 and 2019, we characterized 25 different land cover transition patterns that occurred between 2003 and 2019. These new variables allowed us to separate land cover that had remained stable during this period from newly formed land cover. We estimated the area of each land cover transition pattern. All variables are shown in Table 1.

**Table 1:** Description of variables and their use in the models. The area is defined as the 2500 m buffer zone.

Variable category	Variable	Description	Used in models
Dependent variables	Scrub_cases	Number of scrub typhus cases per year and per village	GAM* land cover (1)**
	Scrub_tot	Number of cumulated scrub typhus cases per village	GAM land cover transition (2)***
Spatio-temporal variables	Year	Year of the variable record	GAM land cover (1)
	Lon	Longitude of the village	GAM land cover (1) and GAM land cover transition (2)
	Lat	Latitude of the village	GAM land cover (1) and GAM land cover transition (2)
Topographic variables	Elevation	Elevation of the village in m	GAM land cover (1)
	Slope_mean	Mean slope of the buffer zone of each village	GAM land cover (1)
Demographic variables	N_men	Number of men per village	GAM land cover (1) and GAM land cover transition (2)
Land cover variables	Rainfed Cropland cover	Yearly area of rainfed cropland cover	GAM land cover (1)
	Grassland cover	Yearly area of grassland cover	
	Mosaic cover	Yearly area of mosaic cover	
	Irrigated cropland cover	Yearly area of irrigated cropland cover	
	Broadleaf forest cover	Yearly area of broadleaf forest cover	
	Urban cover	Yearly area of urban cover	
	Shrubland cover	Yearly area of shrubland cover	
	Needleleaf forest cover	Yearly area of needleleaf forest cover	
	Water cover	Yearly area of water cover	
Land cover transition variables	Irrigated cropland to Mosaic	Area of irrigated cropland that became mosaic	GAM land cover transition (2)
	Stable rainfed cropland	Area of rainfed cropland that has been stable	
	Rainfed cropland to urban areas	Area of rainfed cropland that became urban areas	
	Rainfed cropland to shrubland	Area of rainfed cropland that became shrubland	
	Rainfed cropland to mosaic	Area of rainfed cropland that became mosaic	
	Rainfed cropland to broadleaf forest	Area of rainfed cropland that became broadleaf forest	
	Grassland to shrubland	Area of grassland that became shrubland	
	Grassland to mosaic	Area of grassland that became mosaic	
	Grassland to broadleaf forest	Area of grassland that became broadleaf forest	
	Stable shrubland	Area of shrubland that has been stable	
	Shrubland to irrigated cropland	Area of shrubland that became irrigated cropland	
	Shrubland to rainfed cropland	Area of shrubland that became rainfed cropland	
	Shrubland to grassland	Area of shrubland that became grassland	
	Shrubland to mosaic	Area of shrubland that became mosaic	
	Shrubland to broadleaf forest	Area of shrubland that became broadleaf forest	
	Shrubland to needleleaf forest	Area of shrubland that became needleleaf forest	
	Stable mosaic	Area of mosaic that has been stable	
	Mosaic to shrubland	Area of mosaic that became shrubland	
	Mosaic to broadleaf forest	Area of mosaic that became broadleaf forest	
	Mosaic to needleleaf forest	Area of mosaic that became needleleaf forest	
	Stable broadleaf forest	Area of broadleaf forest that has been stable	
	Broadleaf forest to shrubland	Area of broadleaf forest that became shrubland	
	Broadleaf forest to mosaic	Area of broadleaf forest that became mosaic	
	Stable needleleaf forest	Area of needleleaf forest that has been stable	
	Needleleaf forest to mosaic	Area of needleleaf forest that became mosaic	

The study and transition periods are between 2003 and 2019.

*GAM = Generalized Additive Model.

** Referring to model number (1) described in the statistical analysis section.

***Referring to model number (2) described in the statistical analysis section.

### Statistical analysis

Descriptive analyses were conducted using R software [25] to explore the land cover and spatial distribution and evolution of scrub typhus. A matrix of change was used to examine the land cover change between 2003 and 2019. We identified the villages that overcame land cover changes within the 2500 m buffer zone and the villages that did not. A Mann-Whitney-Wilcoxon non-parametric test was performed to compare the difference in cumulative scrub typhus cumulated cases between these two groups.

We assessed the spatial autocorrelation of scrub typhus cases using semi-variograms. We performed spatial interpolation of cases using Gaussian process regression (kriging) with the *gstat* package v 2.1-1 [28] and *sp* package v1.6-0 [29].

Based on these preliminary analyses, we performed two General Additive Models (GAM) using the *mgcv* package v1.9-1 [30]. The first considered the spatiotemporal land cover dynamics to explain scrub cases per village and per year. This model included the nine land cover area classes, the number of males per village and topographic variables (Table 1). The structure of the tested GAM is given by the following formula:


$$scrub_{cases}=\beta_{\text{0}}+f_{year}\left(Year\right)+f_{n\_men}\left(n_{men}\right)+f_{LonLat}\left(lon,lat\right)+f_{\text{1}}\left(x_{\text{1}}\right)+\ldots +f_{\text{1}\text{1}}\left(x_{\text{1}\text{1}}\right)$$ (1)

Where β0 is the intercept, *f* the unrestricted splines and x1 to x11 were the nine land cover plus the two topographic variables to select, described in Table 1.

We used the number of males per village in the model because scrub typhus affects mainly males than females in Thailand for the period 2001–2018 [22,31]. The sex ratios between our dataset and Thailand are comparable for this period of time (census of 2010) with respectively 98.9 males to 100 females compared to 96.2 males to 100 females [32]. Additional models with the whole population are available in Supporting information S2 Fig. We set a negative binomial function link based on the assessment of the distribution of scrub typhus cases using *fitdistrplus* package v1.1-11 [33]. All land cover and topographic variables were scaled. We adopted a parsimonious approach to select the final model, based on the AIC indices and we checked model for validity. The basic model considered the relationship between scrub typhus, village population (i.e., number of males) to control for the demographic effect, year to control for the temporal autocorrelation as well as special meteorological events, and longitude and latitude to control for spatial autocorrelation revealed in the previous steps of the analysis. As latitude and longitude interaction within a GAM may introduce spatial confounding, we implemented the spatial+ method described in [34] to account for this bias. Models presented in this article are issued from this method. We tested the explanatory factors one by one, selecting the factors that caused the AIC index to drop the most (See Supporting information S3 Fig. for the results of this selection). Concurvity between variables was then checked, variables were excluded when superior to a threshold of 0.8. We used *mgcv* package v1.9-1 [30] and *DHARMa* package v0.4.6 [35] to test the validity of the models and the concurvity. Finally, we checked the quality of the spatiotemporal autocorrelation using ACF plot, the semi-variogram and the maps of residuals.

The second GAM model aimed to explain the cumulative cases of scrub typhus between 2003 and 2019 by the global land cover change in the 2500 m buffer zone that occurred during the same period. The explicative variables included the 25 types of land cover change area, the male population and the interaction between longitude and latitude to account for spatial autocorrelation. The structure of this second GAM was given as follow:


$$\text{scru}b_{tot}=\beta_{\text{0}}+f_{n_{m}en}\left(n_{men}\right)+f_{LonLat}\left(lon,lat\right)+f_{\text{1}}\left(x_{\text{1}}\right)+\ldots +f_{\text{2}\text{5}}\left(x_{\text{2}\text{5}}\right)$$ (2)

Where β0 is the intercept, *f* the unrestricted splines and x1 to x25 were the 25 land cover transitions and topographic variables to select, described in Table 1.

The family was defined as negative binomial, and we also used a parsimonious approach to select the best model based on the AIC indices. Concurvity was also checked following the same method. Once the GAM model was defined, we replaced the unidentified splines with a polynomial function ranging from 0 to 4 degrees to assess the mathematical equation of the effects.

## Results

### Burden of scrub typhus in Nan province

**Fig 2:**
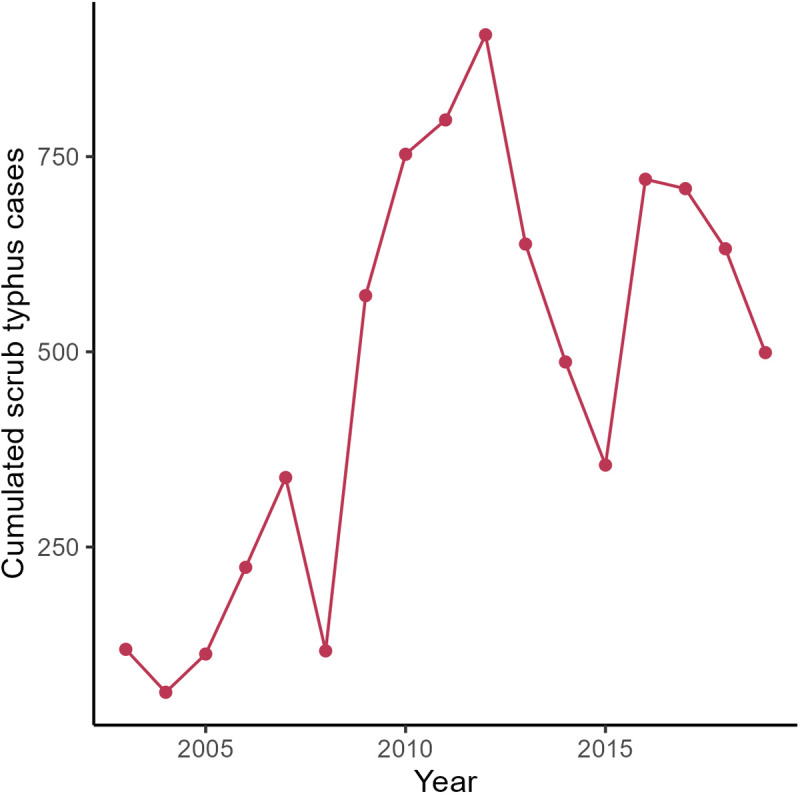
Description of scrub typhus human cases: trend of scrub typhus cases per year.

**Fig 3:**
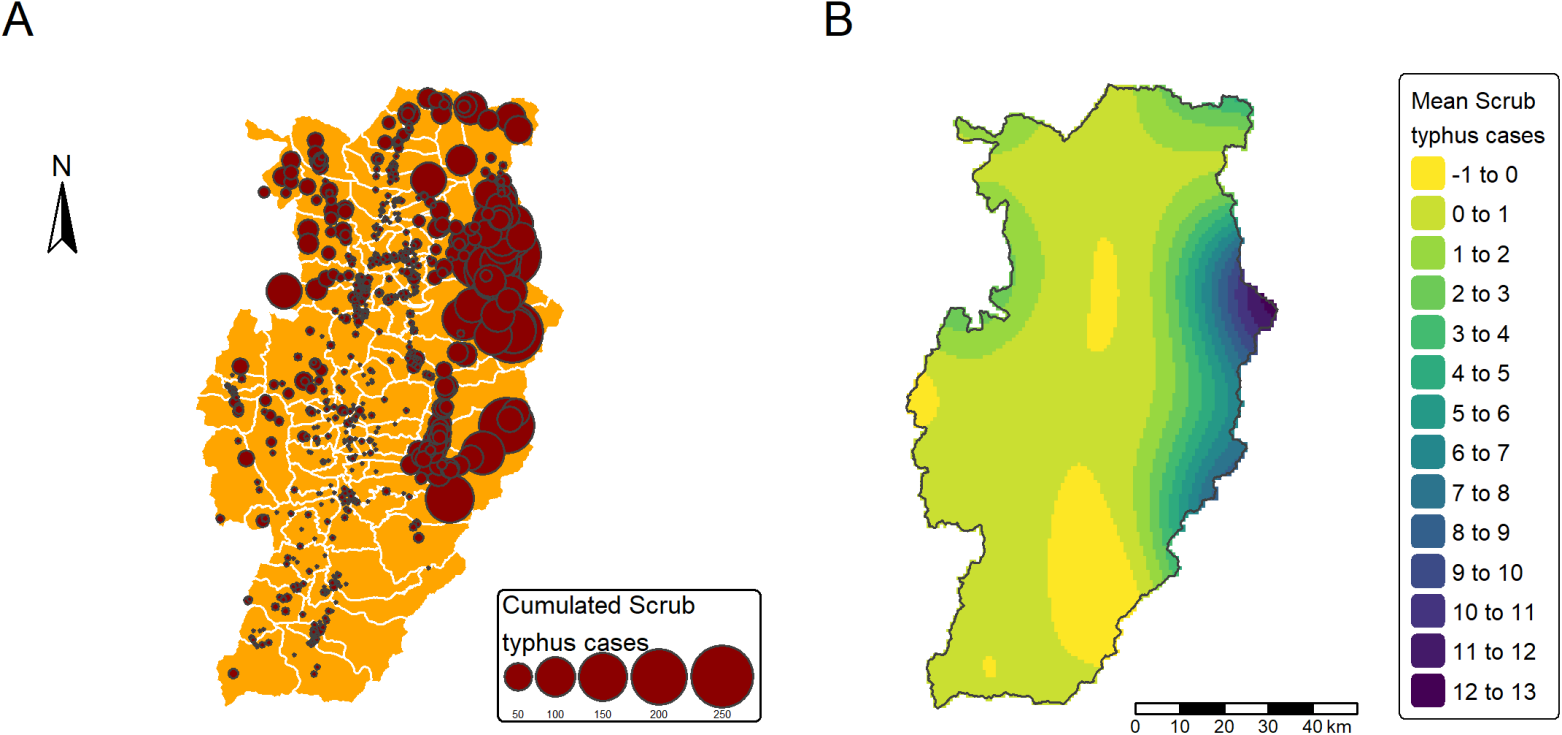
Maps of Nan Province: (A) spatial distribution of cumulative scrub typhus cases between 2003 and 2019 and (B) interpolation of mean scrub typhus cases at village level between 2003 and 2019 by kriging using a semi-variogram based on the village position. The base layer map for administrative boundaries is available at https://data.humdata.org/dataset/cod-ab-tha.

Between 2003 and 2019, a total of 8,045 cases of scrub typhus were referenced by the passive health surveillance system. The burden of scrub typhus was low in 2003 with 119 cases and increased until 2019 (499 cases), reaching two peaks in 2012 (with a number of scrub typhus cases of 906) and 2016 (with a number of scrub typhus cases of 721) (Fig 2). The number of cases per village and per year followed a negative binomial distribution and were spatially heterogeneously distributed. As show in Fig 3A and 3B, the burden of scrub typhus infection between 2003 and 2019 was rather low in the area of Nan River valley, with most cases located in the mountainous and foothill areas of the Northwest, North and East of the valley.

### Land cover within the 2,500 m buffer zone

Nan Province has overcome several land cover changes during the period 2003–2019 (see Supporting information S4 Fig). The province is a rural mountainous area mainly composed of mosaic land (median = 4,203 km²), broadleaf forest (median = 3,249 km²), cropland rainfed (median = 2,394 km²) and shrubland (median = 1,998 km²) between 2003 and 2019. Mosaic land was also predominant within the 2,500m buffer zones followed by cropland rainfed, broadleaf forest and shrubland.

**Fig 4:**
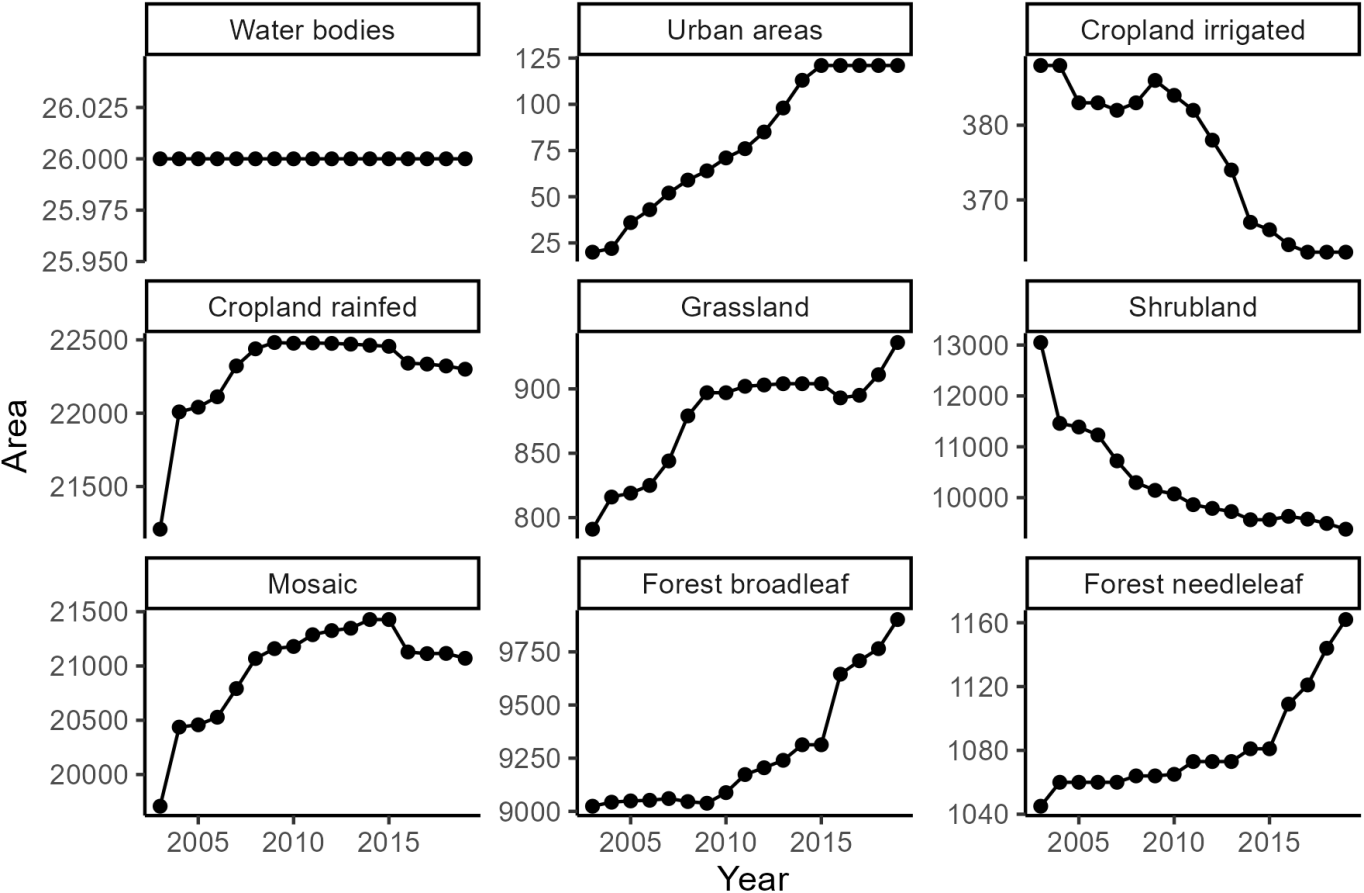
Change in land cover surface area between 2003 and 2019 per land cover class. The y-axes represent the number of cells, which is a square area of 300 meters by 300 meters.

The Fig 4 depicts the land cover changes. The area of rainfed cropland increased from 2003 to 2008 and reached a plateau. Irrigated cropland decreased overall, with an acceleration from 2010. Grassland increased overall and reached a temporary stable area between 2009 and 2017. Broadleaf and needleleaf forests started to increase in 2009 and 2010, respectively. Mosaic land increased until 2015 and started to decrease slightly thereafter. Finally, shrubland decreased sharply until 2019. Water body areas did not change. Finally, urban areas increased and reached a stable area in 2015.

Spatiotemporal effect of land cover on scrub typhus: GAM model results.

**Fig 5:**
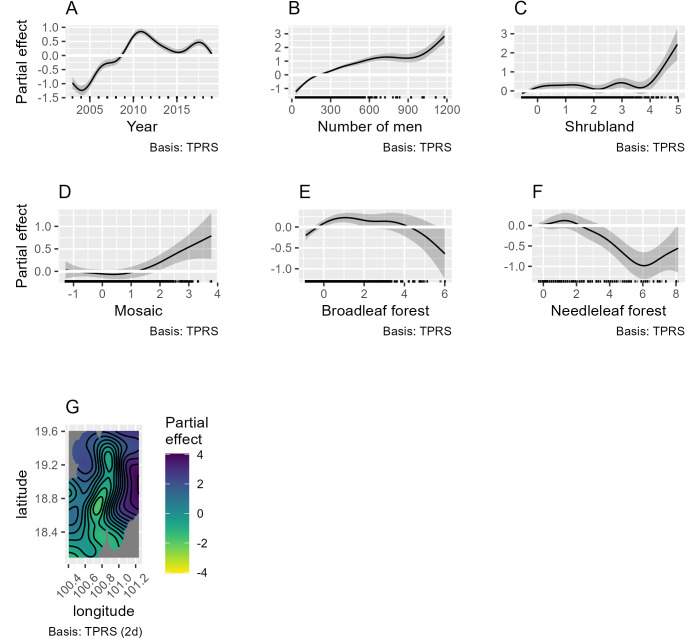
General Additive Modeling (GAM) (1) results of the selected land covers explaining the number of scrub typhus cases in Nan Province between 2003–2019 by villages and year, using a binomial negative link function (theta = 1.075). The smoothed variables selected in the best GAM were: (A) year, (B) number of males per village, (C) shrubland cover, (D) mosaic land cover, (E) broadleaf forest cover, (F) needleleaf forest cover and (G) geographical distribution of villages (longitude and latitude).

A first GAM model (1) was developed considering the yearly scrub typhus cases per village according to the nine yearly land cover surface areas within the 2,500 m buffer zones. The best model was selected according to the Akaike Information Criteria AIC. Intermediate models and their respective AICs are presented in the process of selection described in the Supporting information S3 Fig. The best model explained 64.3% of the deviance (pseudo-R^2^ = 0.54). Rainfed cropland and elevation were excluded from the model due to concurvity problem. The land covers that were significantly associated with the number of scrub typhus cases per village were mosaic land, broadleaf forest (deciduous forest), needleleaf forest and shrubland (Fig 5 and Table 2). The number of scrub typhus cases increased with shrubland and mosaic land areas in the village buffer zones. An inverted U-shape relationship was observed between scrub typhus cases and broadleaf forest area and a negative relationship was observed with needleleaf forest. Mapping of semi-variogram and ACF of the residuals (see Supporting information S5 Fig) showed that the GAM model controlled well for spatial and temporal autocorrelation of scrub typhus cases. The diagnostic of the GAM model (1) is given in Supporting information S6 Fig.

**Table 2:** Results of general additive modelling (GAM) (1) explaining the number of cases of scrub typhus per village in Nan Province using a negative binomial link (theta = 1.075), with approximate significance of smooth terms.

Explanatory variables	Estimated degrees of freedom	Chi square	P-value
S(Year)	8.557	708.32	<2e-16 ***
S(Mosaic)	3.016	22.50	1.61e-04***
S(ForestB)	4.310	34.59	3.04e-06***
S(Shrubland)	7.362	60.04	<2e-16***
S(ForestN)	4.762	49.38	<2e-16***
S(Lon, Lat)	28.177	2515.86	<2e-16***
S(N_men)	6.671	5963.26	<2e-16***

For the best selected model, the deviance explained = 64.3%, R^2^ = 0.54, restricted maximum likelihood (REML) = 8771.8, AIC = 17366.

### Land cover change in Nan province between 2003 and 2019

**Fig 6:**
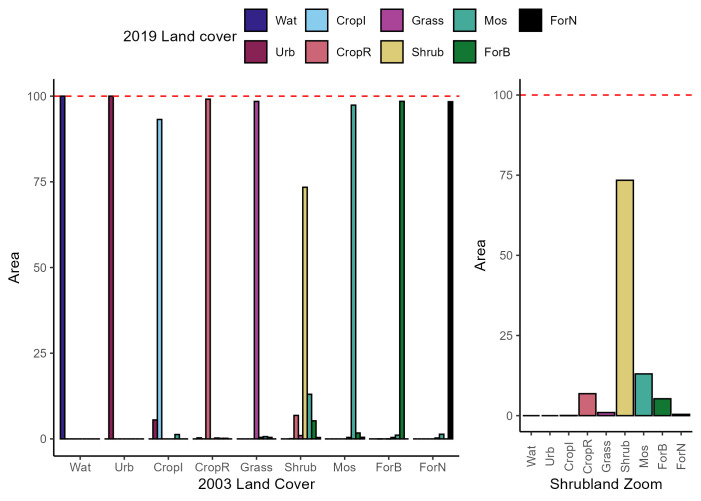
Land cover change matrix of Nan Province between 2003 and 2019. **“**Wat” = Water area, “Urb” = Urban area, “CropI” = Irrigated cropland, “CropR” = Rainfed cropland, “Grass” = Grassland, “Shrub” = Shrubland, “Mos” = Mosaic, “ForB” = Broadleaf forest, “ForN” = Needleleaf Forest. The coloured bar represents the proportion of land cover of 2019 compared to the 2003 period. For example, Water surface area had not changed and still represents 100% of its surface area of 2003. A 100% of the urbanised areas in 2003 stayed urbanised in 2019, however urban areas also expanded on the cropland irrigated field.

The land cover change matrix (Fig 6) showed that Nan Province was in a process of forest encroachment between 2003 and 2019. By 2019, shrubland cover had decreased by 25% of its initial in 2003. This area had been colonized by mosaic land first, then by rainfed cropland followed by broadleaf forest.

The study of the land cover transition model helps to understand the contribution of land cover to explain scrub typhus cases. Here, we considered only the land cover transition of interest for scrub typhus, identified in the first GAM model (1) as: rainfed cropland (at first selected but then excluded because of concurvity problem), mosaic land, broadleaf forest (deciduous forest), needleleaf forest and shrubland. Among the 905 villages, 580 of them experienced significant land cover changes between 2003 and 2019.

### Impact of specific land cover transition on scrub typhus

Villages were categorized as whether they had experienced or not a land cover transition for the five relevant land cover classes. The non-parametric Mann-Whitney-Wilcoxon test showed that villages that overcame relevant land cover changes had more scrub typhus cases than stable villages (median of 2 compared to 1, p-value <0.05).

### Association between cumulative scrub typhus cases and detailed trajectory of land cover change: GAM model results

**Fig 7:**
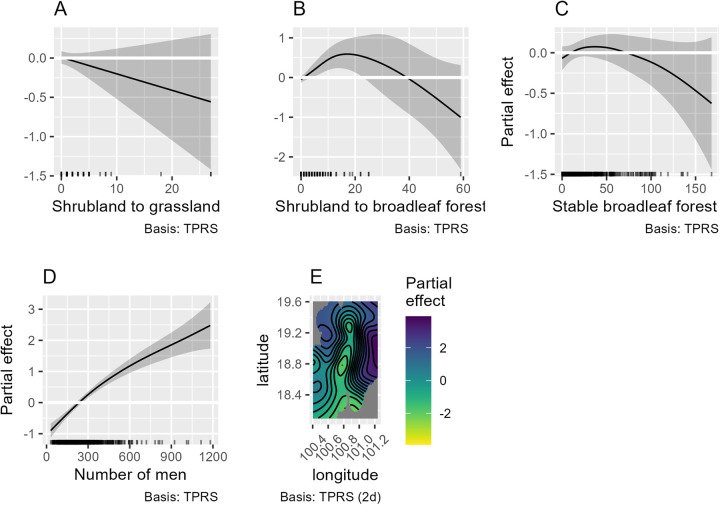
Results of General Additive Modeling (GAM) (2) of selected land cover transition explaining the total number of scrub typhus cases in Nan Province between 2003–2018 at the village level, using a binomial negative link function (theta = 2.74). The smoothed variables selected in the best GAM were: (A) transition from shrubland to grassland, (B) transition from shrubland to broadleaf forest, (C) stable broadleaf forest, (D) the number of males per village and (E) geographical distribution of villages (longitude and latitude).

A second GAM model (2) was developed considering the cumulative scrub typhus cases between 2003 and 2019 per village according to the 25 land cover transitions previously identified in the buffer zones between 2003 and 2019. The best model selected according to AIC is shown in the Table 3 and Fig 7, using a negative binomial linked function (theta = 2.74), with an explained deviance of 81.6% (pseudo-R² of 0.71). The selected variables were stable broadleaf forest, shrubland to grassland change, shrubland to broadleaf forest change, as well as the number of males by village and the longitude and latitude interaction term. Stable rainfed cropland and the transition from rainfed cropland to urban areas were at first selected but then excluded because of concurvity problem. The model (2) diagnostic is given in the Supporting information S7 Fig. The Table 4 shows the fitted polynomial equations for each variable. The GAM output with polynomial functions and its diagnostic are available in the Supporting Information S8 Fig.

**Table 3:** Results of general additive modelling (GAM) (2) of land cover transition explaining the number of cumulative scrub typhus cases per village in Nan Province using a negative binomial link (theta = 2.74), with approximate significance of smooth terms.

Explanatory variables	Estimated degrees of freedom	Chi square	P-value
S(N_men)	2.501	189.027	<2e-16***
S(ForestB_stable)	2.487	4.251	0.236
S(Shrubland_Grassland)	1.002	1.619	0.204
S(Shrubland_ForestB)	2.620	15.287	0.002**
S(Lon,Lat)	26.997	1589.591	<2e-16***

For the best selected model, the deviance explained = 81.6%, R^2^ = 0.71, restricted maximum likelihood (REML) = 1408.9, AIC = 2738.

**Table 4:** Results of general additive modelling (GAM) of land cover transition explaining the number of cumulative cases of scrub typhus per village in Nan Province using a negative binomial link (theta = 2.712), with polynomial adapted terms.

Explanatory variables	Estimate	Std Error	z-value	P-value
(Intercept)	-0.062	0.153	-0.402	0.6877
poly(N_men, 2, raw = T)1	0.005	6.578e-04	7.434	1.05e-13***
poly(N_men, 2, raw = T)2	-1.766e-06	6.840e-07	-2.581	0.0098**
poly(ForestB_stable, 3, raw = T)1	0.012	7.061e-03	1.765	0.07756
poly(ForestB_stable, 3, raw = T)2	-1.732e-04	1.283e-04	-1.350	0.17687
poly(ForestB_stable, 3, raw = T)3	5.054e-07	6.203e-07	0.815	0.41522
Shrubland_Grassland	-0.018	0.016	-1.076	0.28197
poly(Shrubland_ForestB, 3, raw = T)1	0.080	0.032	2.460	0.01391*
poly(Shrubland_ForestB, 3, raw = T)2	-2.753e-03	2.395e-03	-1.150	0.25033
poly(Shrubland_ForestB, 3, raw = T)3	1.859e-05	3.276e-05	0.567	0.57038

For the best selected model, the deviance explained = 81.5%, R^2^ = 0.662, restricted maximum likelihood (REML) = 1466.1, AIC = 2736.766.

As shown in Fig 7, stable broadleaf forest area was not a significant risk factor. However, the reforestation process behind the transition from shrubland to broadleaf forest showed an inverted U-shape relationship with scrub typhus human cases. The relationship shows an initial increase at low levels of reforested area, followed by a decrease at higher reforested surface values, likely driven by a single data point with substantial reforestation and greater uncertainty. A polynomial function of degree 3 was found to give the best fit. Loss of shrubland to grassland was not significant (coefficient = - 0.018, p-value = 0.28197 in the fitted GAM-polynomial model). Male population was positively correlated with the cumulative scrub typhus cases (polynomial function of degree 2).

## Discussion

### Summary of main results

We investigated several environmental factors associated with scrub typhus cases at the village level. First, we confirmed the effect of topography, with relatively few cases in the Nan River valley and most cases in villages localized at higher altitude in mountainous and foothill areas (although slope was not selected in the best model). Our results support our first hypothesis that forest cover was related to scrub typhus cases, but with slight differences. Shrubland and mosaic land were the most important land cover types associated with scrub typhus. Interestingly, needleleaf forest appeared to reduce the risk. Broadleaf forest cover showed an inverted U-shape relationship with scrub typhus cases, suggesting that a very small surface or a large area of forest cover surrounding a village was associated with a lower risk of scrub typhus transmission. By studying land cover dynamics and transitions, we showed that broadleaf forest cover, which had remained stable over the period, was no longer an important factor in scrub typhus transmission. Conversely, the reforestation process identified in the transition from shrubland to forest appeared significantly at risk for scrub typhus. Villages that experienced land cover changes during the period 2003–2019 also recorded significantly higher scrub typhus cases than stable villages. However, these stable villages were not evenly distributed in Nan Province and were located in agrarian valley rather than in foothill and mountainous areas. These results support our second hypothesis that unstable land covers such as secondary growth vegetation or fragmented landscape may increase the burden of scrub typhus compared to stable land covers. The spatial interaction term in the models could have introduced spatial confounding [34,36]. We checked for such bias and found that the corrected models presented in the article were highly similar to the initial models (see Supporting Information S9 Fig) which strengthen the explanatory power of the land cover and land cover transition variables.

The scrub typhus vector mite, may not tolerate all types of land cover change. Gain in forest cover, as illustrated by the transition from shrubland to forest, appears to increase the risk of scrub typhus transmission when the surface area is intermediate (i.e., inverted U-shape relationship). Shrubland to grassland change studied in the transition model (2) suggests that less bushy coverage might reduce the transmission of the disease (yet not significant). This may indicate that the vector of scrub typhus requires some tree cover to thrive and may not tolerate too much habitat degradation. The ecological states and the functional processes of forest ecosystems are important for scrub typhus dynamics. Further research on vector ecology is needed to understand how.

The importance of forest cover and vegetation highlighted in this study had been previously observed in several studies in China and Taiwan [15–17,37–39]. Our results are also in accordance with Wardrop et al. [17] and Li et al. [15] who identified transitional land covers and in particular mosaic land and shrubland as significant variables explaining scrub typhus cases. The impact of land cover dynamics on scrub typhus had been poorly studied [39], but the study of Min et al. [18] suggested that scrub typhus thrives with secondary growth vegetation. Min et al. [18] observed this phenomenon in a deforestation context in South Korea based on Global Forest Watch data. Deforestation was not significant in our modelling results although we observed that stable forest was less at risk than newly grown forest from 2003 to 2019. Wangrangsimakul et al. [3] investigated scrub typhus incidence in Chiang Rai Province in Northern Thailand, near Nan Province. Consistent with our study, they also observed a positive association between elevation and scrub typhus. These authors also used land cover data from the European Spatial Agency, though at 1 km resolution. However, in contrast to our results, they observed that forest cover and mosaic land decreased scrub typhus incidence while habitat complexity increased it. These differences could be explained by the differences in scales (subdistrict level versus villages level) and the presence of different variables (such as landscape complexity).

To explain the importance of secondary growth vegetation, further studies should investigate the vectors (chiggers), hosts and pathogen in relation to the environment. Kuo et al. [13] compared rodent infestation by chiggers and ticks in fallow (abandoned fields) and ploughed fields in Taiwan. The burden of chiggers was three times higher in fallows where secondary vegetation provided suitable microhabitats. An early study revealed that in Malaysia in 1974, the distribution of the vector mirrored the distribution of its main host, *Rattus tanemuz*i [40]. This rodent species is generalist and synanthropic and should be favoured in disturbed areas. The importance of this rodent host for the chiggers and the pathogen has been confirmed in more recent studies in Southeast Asia [41] and more specifically in northern Thailand [42]. Network analysis showed that *Rattus tanezumi* with the other synanthropic rodent *Bandicota indica* as well as the chigger species *Leptotrombidium deliense* and *Walchia kritochaeta* emerged as central nodes in rodents-chiggers networks [42]. *Orientia tsutsugamushi* positive chiggers and rodents were more abundant in lowland than in forested areas. However, another study in Thailand found that chigger species richness was higher in forested areas rather than in human-disturbed habitats [6]. A similar study was also conducted in Thailand on the association between human land use and occurrence of *Orientia tsutsugamushi* in rodents [43]. Rodent infected with *Orientia tsutsugamushi* were also more likely to be found in forested habitats. The authors suggested that rodents were likely infected in habitats such as houses, fallow land or rice fields when they were close to forested areas. The results on chiggers and rodents led the authors to suggest that a minimum threshold of biodiversity was required for vectors, hosts and the pathogen. However, these authors also showed that scrub typhus incidence was negatively associated with host-parasite network connectance, suggesting that high complexity of interactions might reduce human exposure, or by giving non-vector species a better chance to dominate. This may be reflected in the inverted U-shaped relationship between broadleaf forest cover and scrub typhus cases. An alternative explanation is that disturbed areas such rainfed cropland, may not provide the microclimate requirements for the vectors [14]. Another local study in Malaysia highlighted the importance of habitat complexity and ecotone as a potential risk factor [44]. In this study, the authors found that chigger species richness on rodents was highest at forest edges compared to four other land cover classes. However, none of the rodents were infected with *Orientia tsutsugamushi* in this habitat, while the highest prevalence was observed in rodents trapped in oil palm plantation. To further support the importance of forest edge and transitional land covers, a previous empirical study in a temperate area (Nebraska) indicated that chiggers were found all along forest edges. But the vectors particularly thrived in short to tall-grass transition zones rather than in the understorey with important tree canopy [45]. Understanding scrub typhus ecology in term of habitat remains very challenging. Further empirical data on vector distribution, combined with detailed habitat description, are needed.

## Limitations

Our studies had several limitations. First, the type of satellite images available showed mixed results. The literature on land use change has shown that Nan Province suffers from deforestation, mainly due to the expansion of maize and commercial tree plantations [46]. These results were not observed using ESA CCI land cover data but using a combination of different satellite images with much higher resolution. One explanation is that what is recognised as mosaic land and shrubland cover in ESA CCI land covers was often considered forest cover in some studies [20,46]. Another explanation is that the rubber plantation cover, which doubled in area between 1990 and 2019 [46], was recognised as forest or mosaic land and not as agricultural land. This leads to the second limitation, commercial plantations such as rubber, teak or orchard are not classified, making it difficult to validate or invalidate the observation of a positive association between scrub typhus cases and commercial plantations [47]. The same problem applies to some crops such as maize, which has increased dramatically between 2003 and 2019. A third limitation is associated with agricultural practices, which make land covers unstable, such as slash-and-burn cultivation, crop rotation with fallow that reduces over the years [48]. Therefore, it remains difficult to study land cover changes in an area with such low resolution, either in area or classification. A fourth limitation concerns the impact of abiotic factors. Abiotic factors such as rainfall and temperature have a significant impact on scrub typhus transmission [49]. Our results showed (Fig 2) that scrub typhus cases increased significantly in 2010 and 2016 during ENSO events [50]. The year variable in GAM model (1) accounted for such climatic variation during the period 2003–2019. However, no data of good quality was available at such a fine resolution. Nan province possesses only two meteorological stations and the prediction of temperature and humidity at the village level did not enabled us to use this data with good satisfactory along the land cover variables. A fifth limitation is the lack of information on the behaviour of populations and their exposure to risky habitats around their villages. Socio-economic and vocation data that could explain such information were not available at the village level which prevented us to use it in the model. Finally, diagnostic limitations and passive surveillance likely led to underreporting. Most hospitals lack confirmatory tests, in reliance on clinical criteria with moderate accuracy. Incomplete reporting and variable care-seeking behaviour further underestimate the true burden.

Choosing the immediate vicinity of the villages as the area of interest allowed us to link local ecological studies on vectors, hosts, and pathogens with regional epidemiological analysis of scrub typhus burden in humans. As sampling the vector when it is free-living in the environment is a high effort with low efficiency, this study represents a further step towards a comprehensive understanding of scrub typhus risk habitats and transmission ecology. In particular, it contributes to the knowledge of the scrub typhus situation in northern Thailand, an endemic area. Understanding the relationship between land cover and human cases offers new opportunities for developing strategies to reduce the human burden of disease. To summarize, our study highlighted tree, shrub, second growth vegetation and unstable land cover as risky habitats where needleleaf forests and broadleaf (deciduous) forests, if stable, may have a protective effect. A higher resolution of the land cover map could refine the conclusions, especially regarding agricultural land and process of forest fragmentation we could not assess here.

## Supporting information

S1 Fig Land use–land cover classification for Nan province. Extracted form Land cover CCI product user guide version 2.0.
(DOCX)

S2 Fig Charts of general additive model (GAM (1)) explaining the number of scrub typhus cases according to land cover surface area, elevation, slope and the number of people (male and female) per villages and GAM (2) explaining the cumulated number of scrub typhus cases according to land cover change surface area, elevation and the number of people (male and female) per villages. (PDF)

S3 Fig Selection of Gam model based on AIC. (PDF)

S4 Fig Maps of Nan Province: (A) land cover in 2003, (B) land cover in 2019, and (C) land cover change between 2003 and 2019 (*LC = land cover). The base layer of the map of administrative boundaries is available at https://data.humdata.org/dataset/cod-ab-tha.
(PDF)

S5 Fig (A) Map of the GAM residuals, (B) ACF plot verifying temporal autocorrelation of GAM land cover residuals, (C) semivariogram of GAM land cover residuals verifying spatial autocorrelation. The base layer of the map is available at https://data.humdata.org/dataset/cod-ab-tha.
(PNG)

S6 Fig Land cover GAM diagnostic. (PDF)

S7 Fig Land cover transition GAM diagnostic. (PDF)

S8 Fig Charts of general additive model (GAM) explaining the number of cumulated cases of scrub typhus per villages in Nan province using a negative binomial link with polynomial adapted terms and its diagnostic. Adapted GAM-polynomial model.
(PDF)

S9 Fig Land cover and land cover transition GAMs and their diagnostic without spatial confounding correction (GAM (1): pages 1&2, GAM (2): pages 3&4). (PDF)
