# Supplementary material for: Scrub typhus in Nan province (Thailand): Seventeen years of data to understand the impact of land cover change
Source: PLoS Negl Trop Dis. 2025 Sep 18;19(9):e0013552. doi: 10.1371/journal.pntd.0013552 (PMC12469158; doi:10.1371/journal.pntd.0013552)
Supplement: S1 Fig — Extracted form Land cover CCI product user guide version 2.0. (DOCX) [file pntd.0013552.s001.docx]

S1 Land use–land cover classification for Nan province.

Extracted from the Land cover CCI product user guide version 2.0 (document ref: CCI-LC-PUGV2; last modified: 2017-04-10)

| VALUE | LABEL |
| --- | --- |
| 0 | No Data |
| 10 | Cropland, rainfed |
| 20 | Cropland, irrigated or post-flooding |
| 30 | Mosaic cropland (>50%) / natural vegetation (tree, shrub, herbaceous cover)  (<50%) |
| 40 | Mosaic natural vegetation (tree, shrub, herbaceous cover) (>50%) / cropland  (<50%) |
| 50 | Tree cover, broadleaved, evergreen, closed to open (>15%) |
| 60 | Tree cover, broadleaved, deciduous, closed to open (>15%) |
| 70 | Tree cover, needleleaved, evergreen, closed to open (>15%) |
| 80 | Tree cover, needleleaved, deciduous, closed to open (>15%) |
| 90 | Tree cover, mixed leaf type (broadleaved and needleleaved) |
| 100 | Mosaic tree and shrub (>50%) / herbaceous cover (<50%) |
| 110 | Mosaic herbaceous cover (>50%) / tree and shrub (<50%) |
| 120 | Shrubland |
| 130 | Grassland |
| 140 | Lichens and mosses |
| 150 | Sparse vegetation (tree, shrub, herbaceous cover) (<15%) |
| 160 | Tree cover, flooded, fresh or brakish water |
| 170 | Tree cover, flooded, saline water |
| 180 | Shrub or herbaceous cover, flooded, fresh/saline/brakish water |
| 190 | Urban areas |
| 200 | Bare areas |
| 210 | Water bodies |
| 220 | Permanent snow and ice |
