# Supplementary material for: Scrub typhus in Nan province (Thailand): Seventeen years of data to understand the impact of land cover change
Source: PLoS Negl Trop Dis. 2025 Sep 18;19(9):e0013552. doi: 10.1371/journal.pntd.0013552 (PMC12469158; doi:10.1371/journal.pntd.0013552)

GAM (1) with the number of people (male and female) per villages.

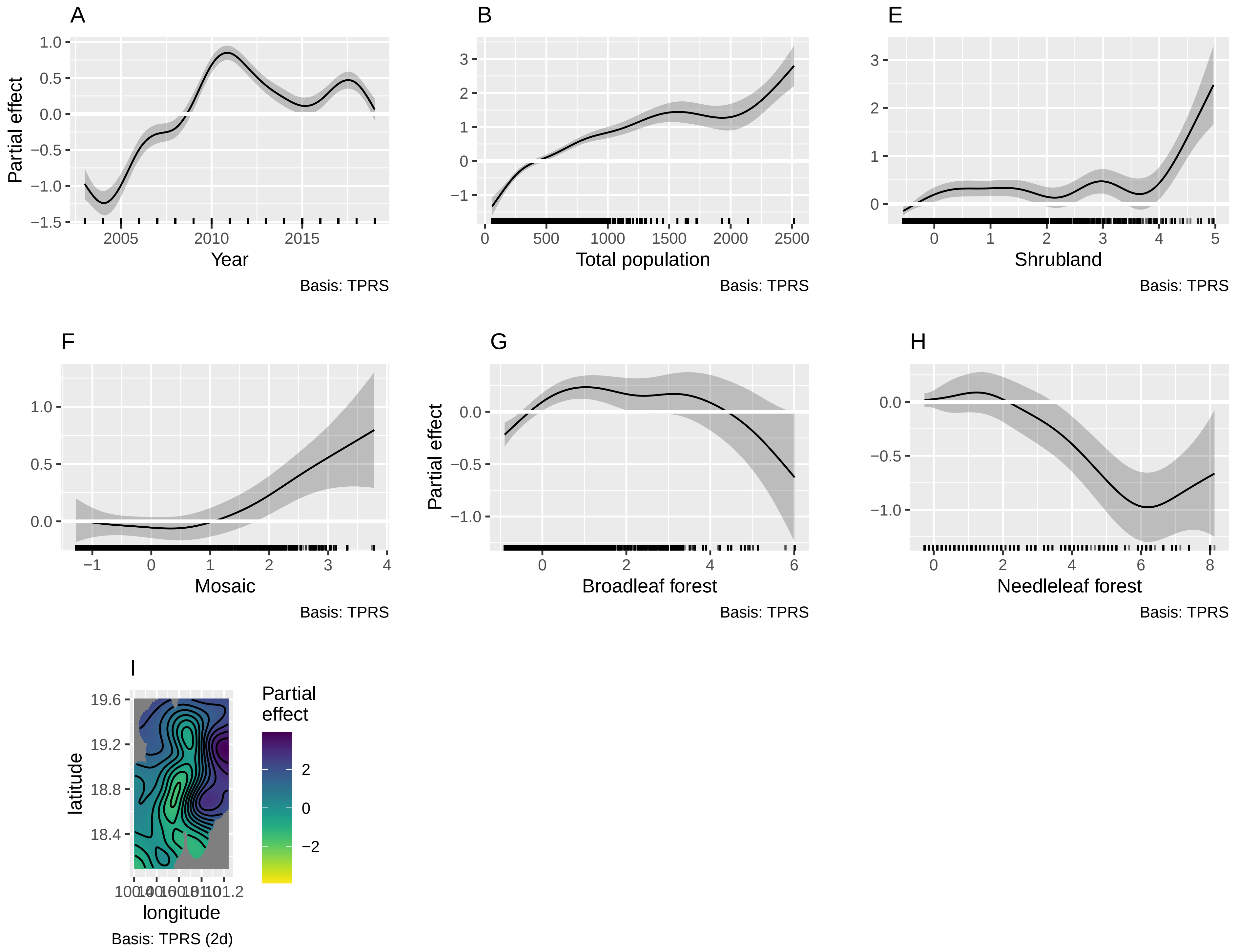

GAM (2) with the number of people (male and female) per villages.

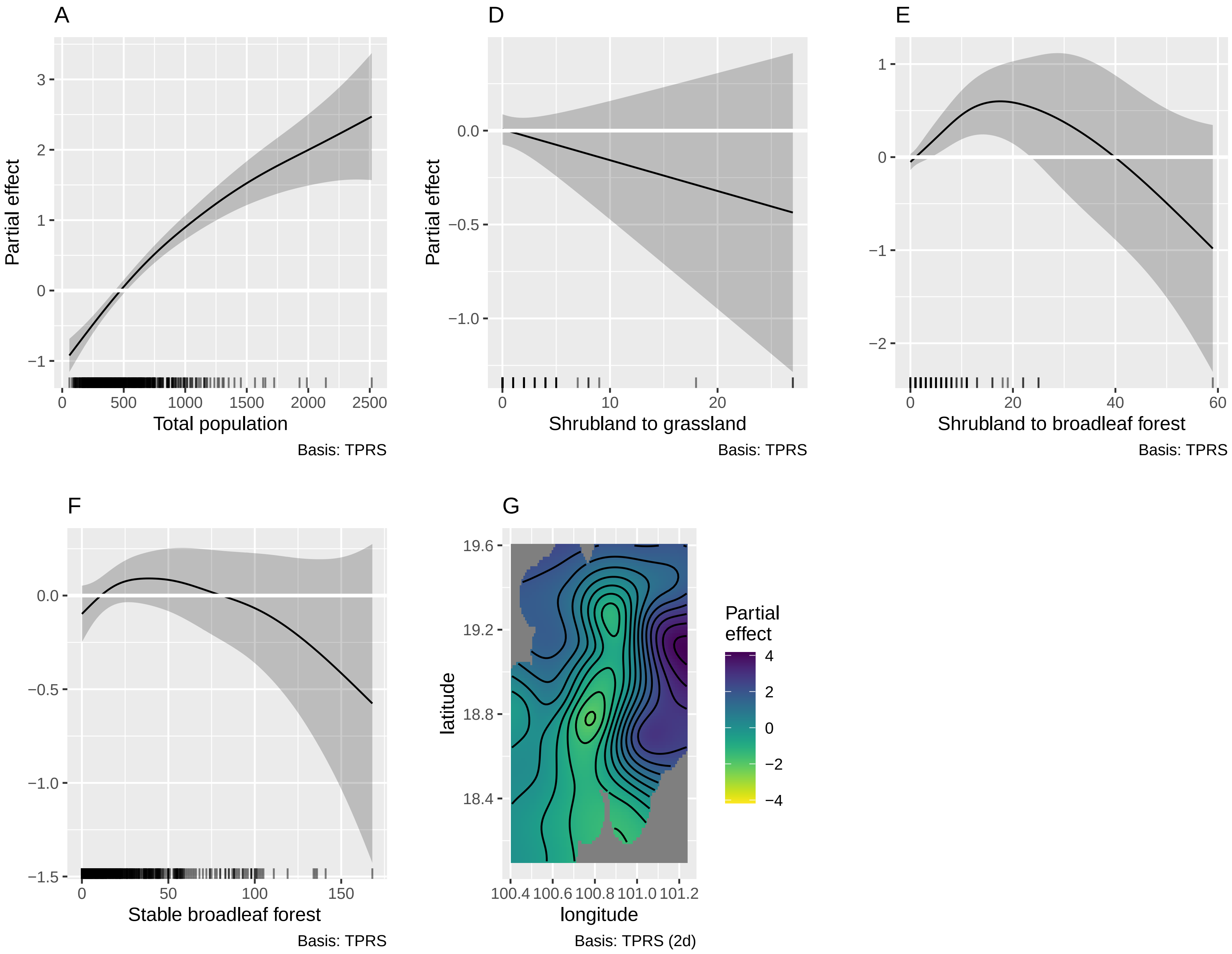

Supplement: S2 Fig — (PDF) [file pntd.0013552.s002.pdf]
