# Supplementary figures and images for: Scrub typhus in Nan province (Thailand): Seventeen years of data to understand the impact of land cover change
Source: PLoS Negl Trop Dis. 2025 Sep 18;19(9):e0013552. doi: 10.1371/journal.pntd.0013552 (PMC12469158; doi:10.1371/journal.pntd.0013552)

# Selection of GAM model based on AIC

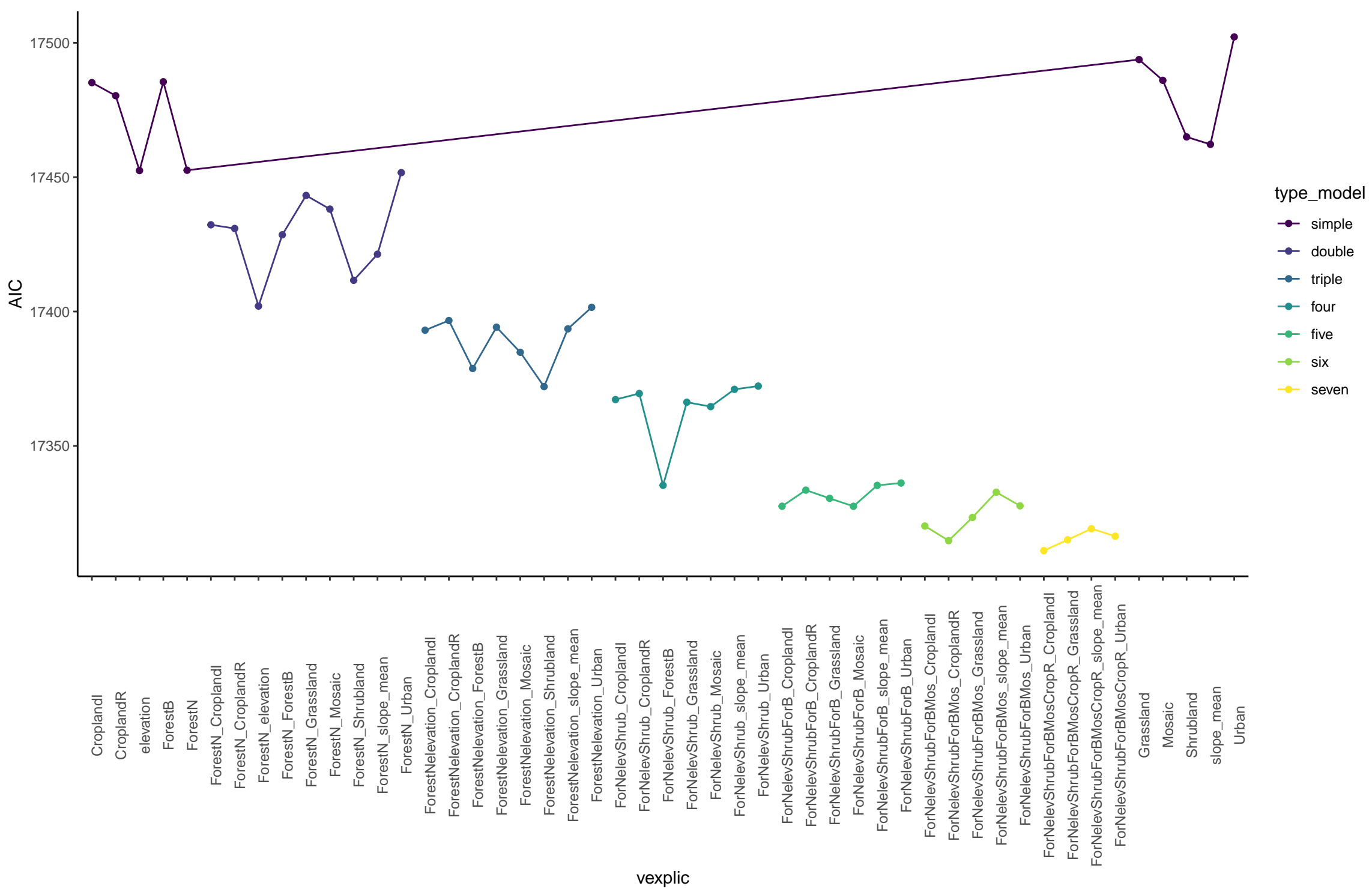

Supplement: S3 Fig — (PDF) [file pntd.0013552.s003.pdf]

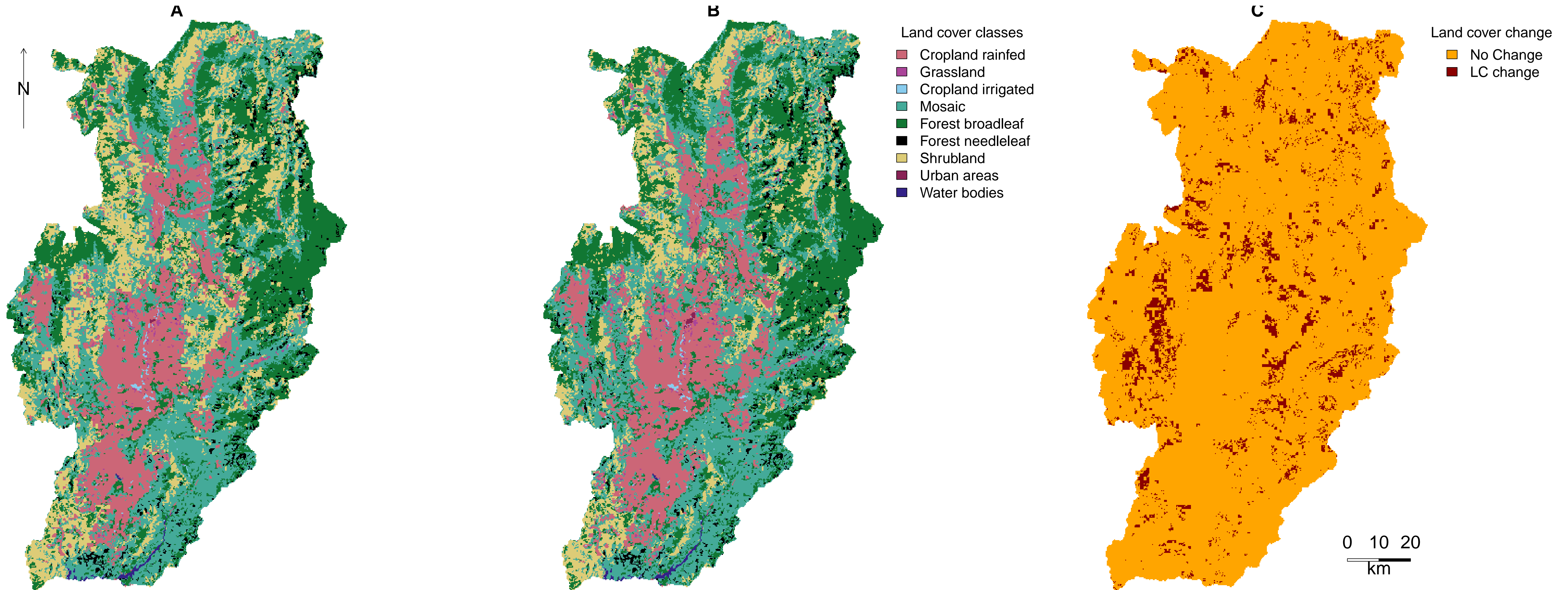

Supplement: S4 Fig — (A) land cover in 2003, (B) land cover in 2019, and (C) land cover change between 2003 and 2019 (*LC = land cover). The base layer of the map of administrative boundaries is available at https://data.humdata.org/dataset/cod-ab-tha. (PDF) [file pntd.0013552.s004.pdf]

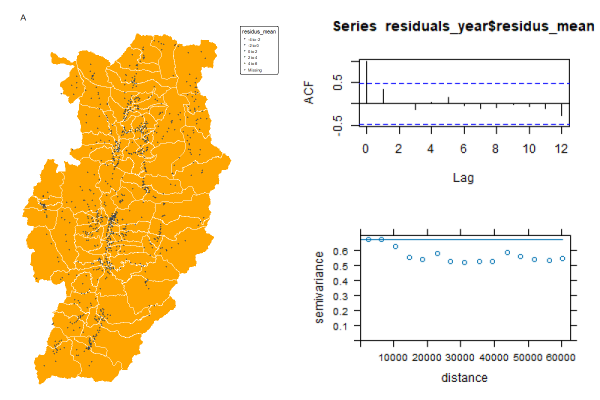

Supplement: S5 Fig — (A) Map of the GAM residuals, (B) ACF plot verifying temporal autocorrelation of GAM land cover residuals, (C) semivariogram of GAM land cover residuals verifying spatial autocorrelation. The base layer of the map is available at https://data.humdata.org/dataset/cod-ab-tha. (PNG) [file pntd.0013552.s005.png]
