# Supplementary material for: Scrub typhus in Nan province (Thailand): Seventeen years of data to understand the impact of land cover change
Source: PLoS Negl Trop Dis. 2025 Sep 18;19(9):e0013552. doi: 10.1371/journal.pntd.0013552 (PMC12469158; doi:10.1371/journal.pntd.0013552)
Supplement: S7 Fig — (PDF) [file pntd.0013552.s007.pdf]

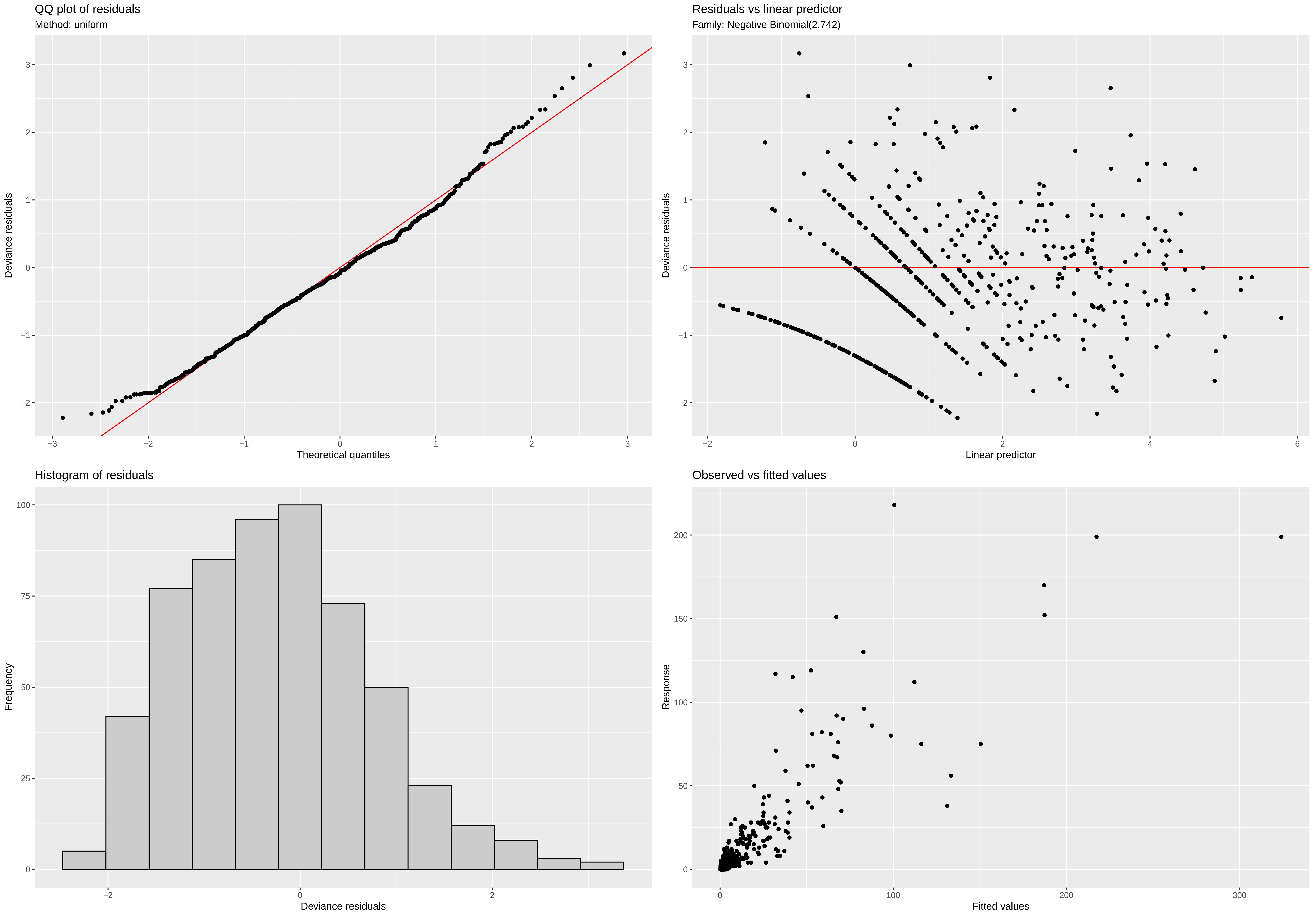
QQ plot of residuals
Method: uniform
Deviance residuals
Theoretical quantiles
Residuals vs linear predictor
Family: Negative Binomial(2.742)
Deviance residuals
Linear predictor
Histogram of residuals
Frequency
Deviance residuals
Observed vs fitted values
Response
Fitted values
