# Supplementary material for: Scrub typhus in Nan province (Thailand): Seventeen years of data to understand the impact of land cover change
Source: PLoS Negl Trop Dis. 2025 Sep 18;19(9):e0013552. doi: 10.1371/journal.pntd.0013552 (PMC12469158; doi:10.1371/journal.pntd.0013552)
Supplement: S8 Fig — Adapted GAM-polynomial model. (PDF) [file pntd.0013552.s008.pdf]

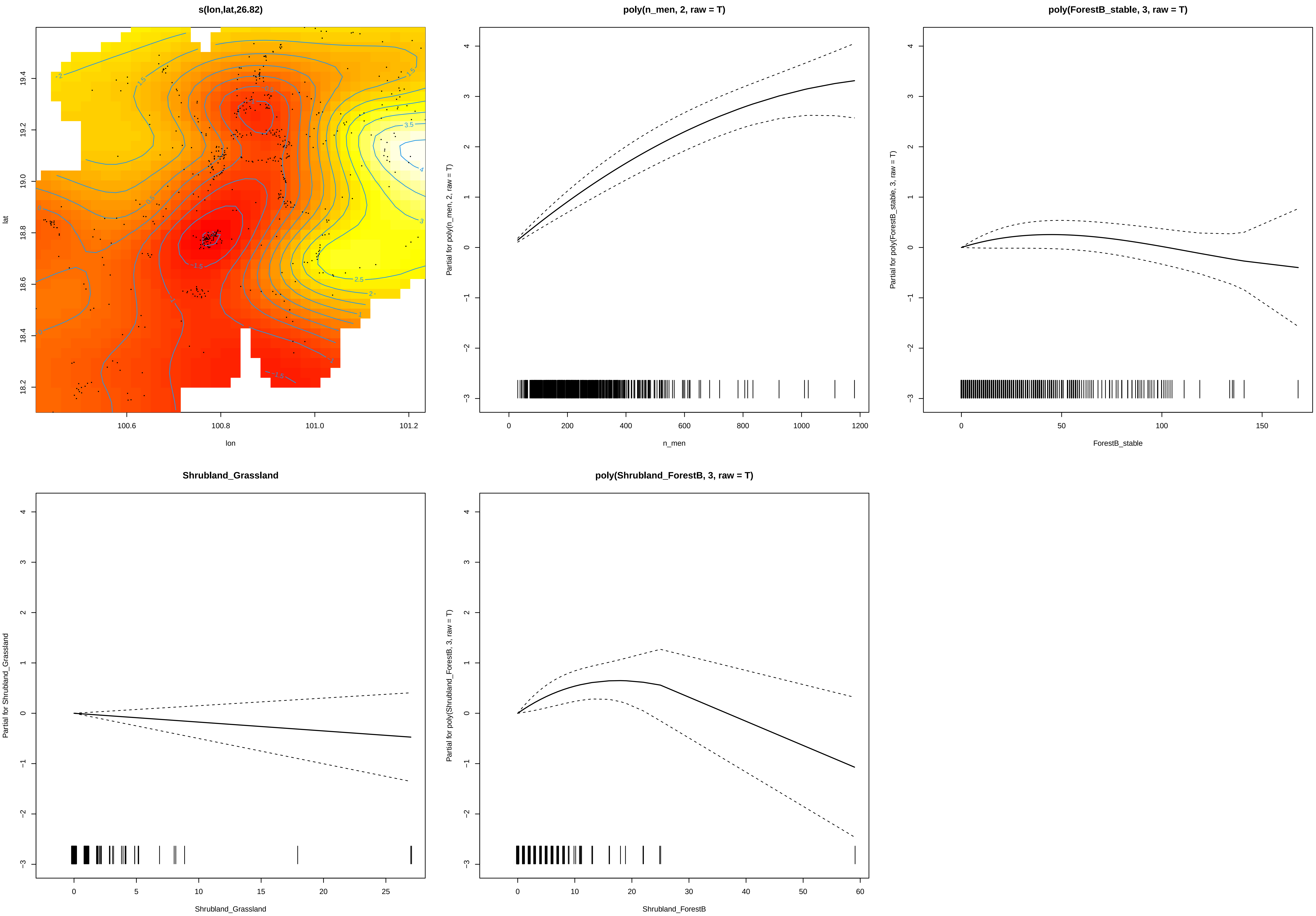
s(lon,lat,26.82)
lat
lon
poly(n_men, 2, raw = T)
Partial for poly(n_men, 2, raw = T)
n_men
poly(ForestB_stable, 3, raw = T)
Partial for poly(ForestB_stable, 3, raw = T)
ForestB_stable
Shrubland_Grassland
Partial for Shrubland_Grassland
Shrubland_Grassland
poly(Shrubland_ForestB, 3, raw = T)
Partial for poly(Shrubland_ForestB, 3, raw = T)
Shrubland_ForestB

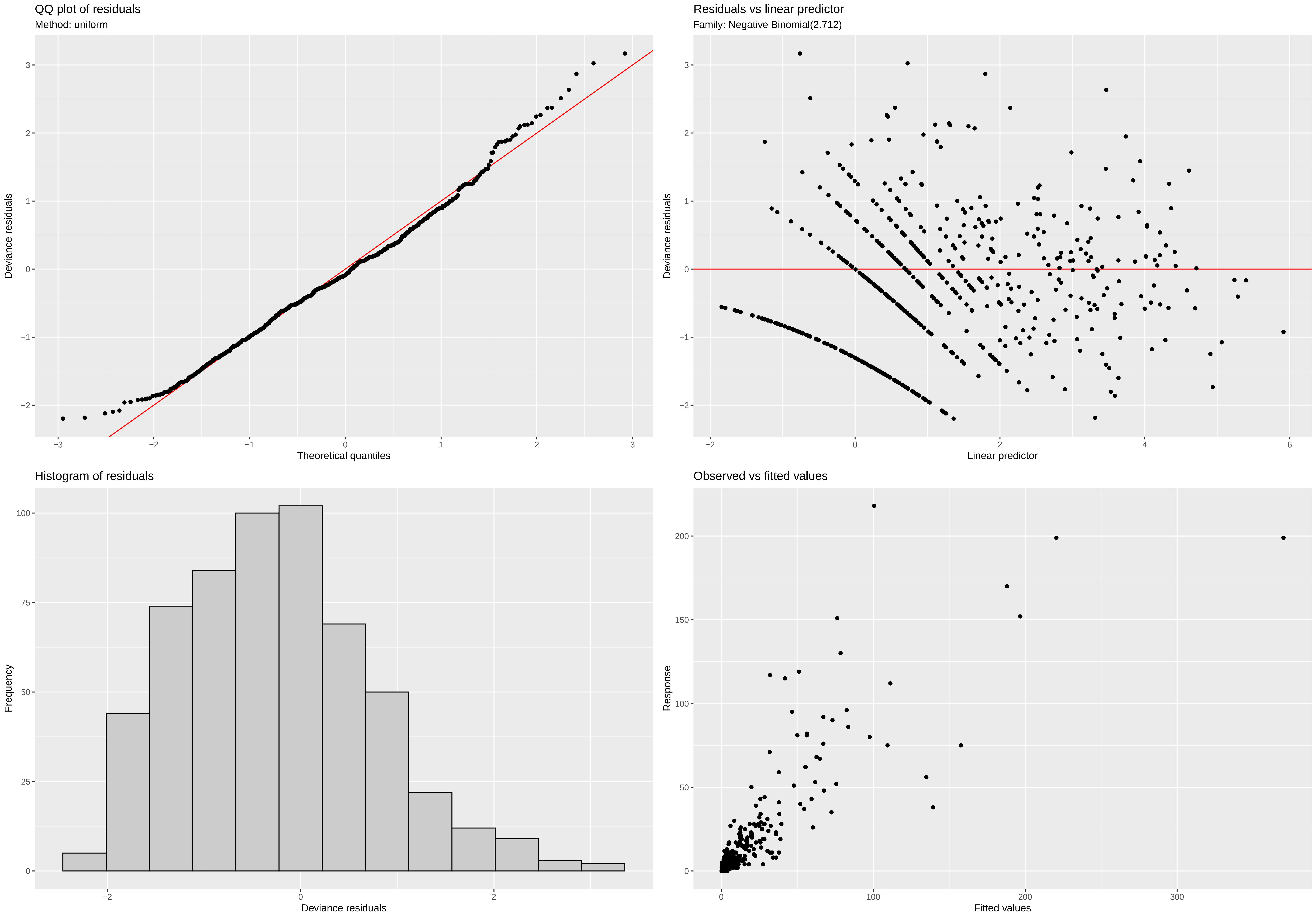
QQ plot of residuals
Method: uniform
Deviance residuals
Theoretical quantiles
Residuals vs linear predictor
Family: Negative Binomial(2.712)
Deviance residuals
Linear predictor
Histogram of residuals
Frequency
Deviance residuals
Observed vs fitted values
Response
Fitted values
