# Supplementary material for: Scrub typhus in Nan province (Thailand): Seventeen years of data to understand the impact of land cover change
Source: PLoS Negl Trop Dis. 2025 Sep 18;19(9):e0013552. doi: 10.1371/journal.pntd.0013552 (PMC12469158; doi:10.1371/journal.pntd.0013552)
Supplement: S9 Fig — (PDF) [file pntd.0013552.s009.pdf]

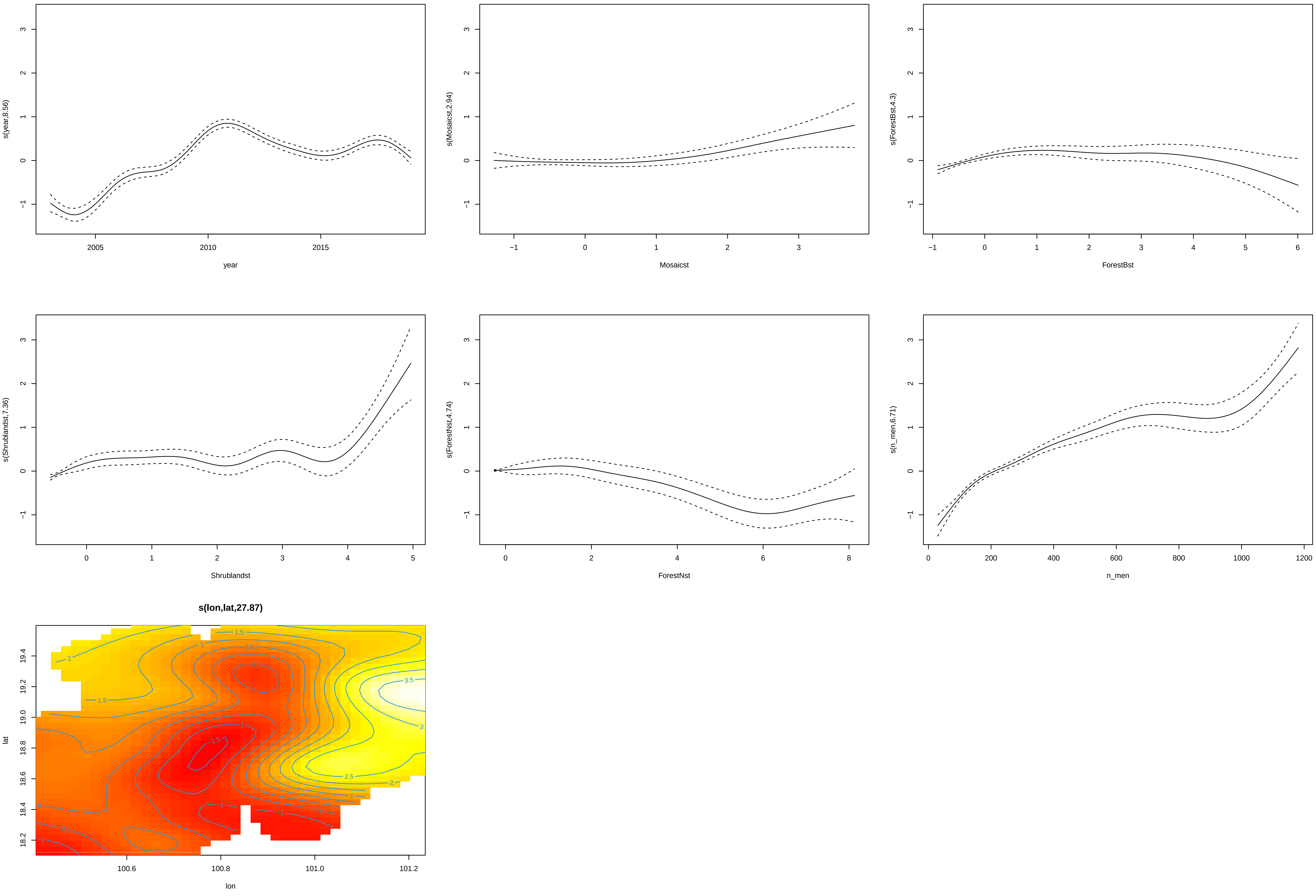

s(year,8.56)
year
2005
2010
2015
s(Mosaicst,2.94)
Mosaicst
s(ForestBst,4.3)
ForestBst
s(Shrublandst,7.36)
Shrublandst
s(ForestNst,4.74)
ForestNst
s(n_men,6.71)
n_men
s(lon,lat,27.87)
lon
lat
100.6
100.8
101.0
101.2
18.2
18.4
18.6
18.8
19.0
19.2
19.4

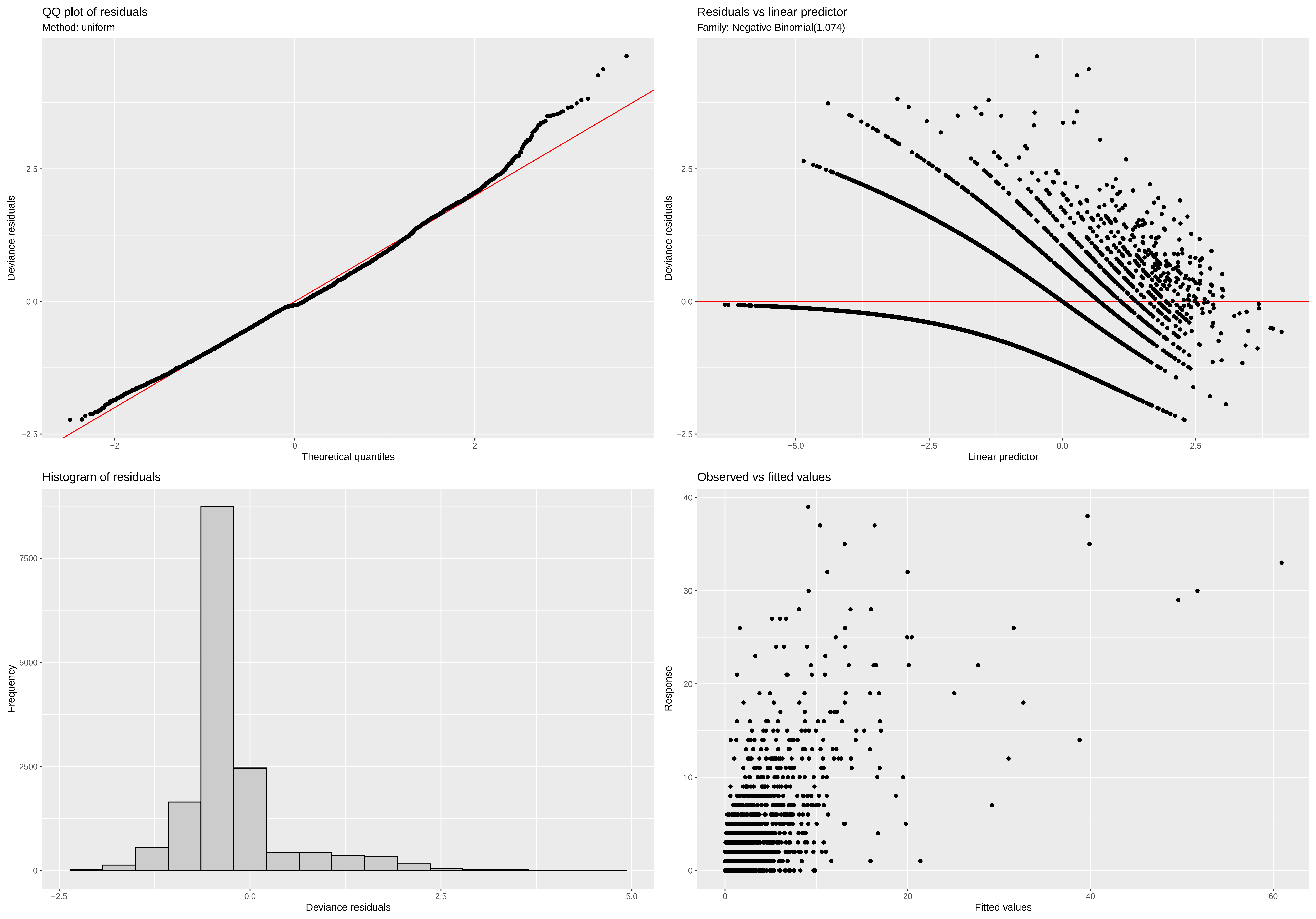
QQ plot of residuals
Method: uniform
Deviance residuals
Theoretical quantiles
2.5
0.0
−2.5
−2
0
2
Residuals vs linear predictor
Family: Negative Binomial(1.074)
Deviance residuals
Linear predictor
−5.0
−2.5
0.0
2.5
Histogram of residuals
Frequency
Deviance residuals
7500
5000
2500
0
−2.5
0.0
2.5
5.0
Observed vs fitted values
Response
Fitted values
40
30
20
10
0
0
20
40
60

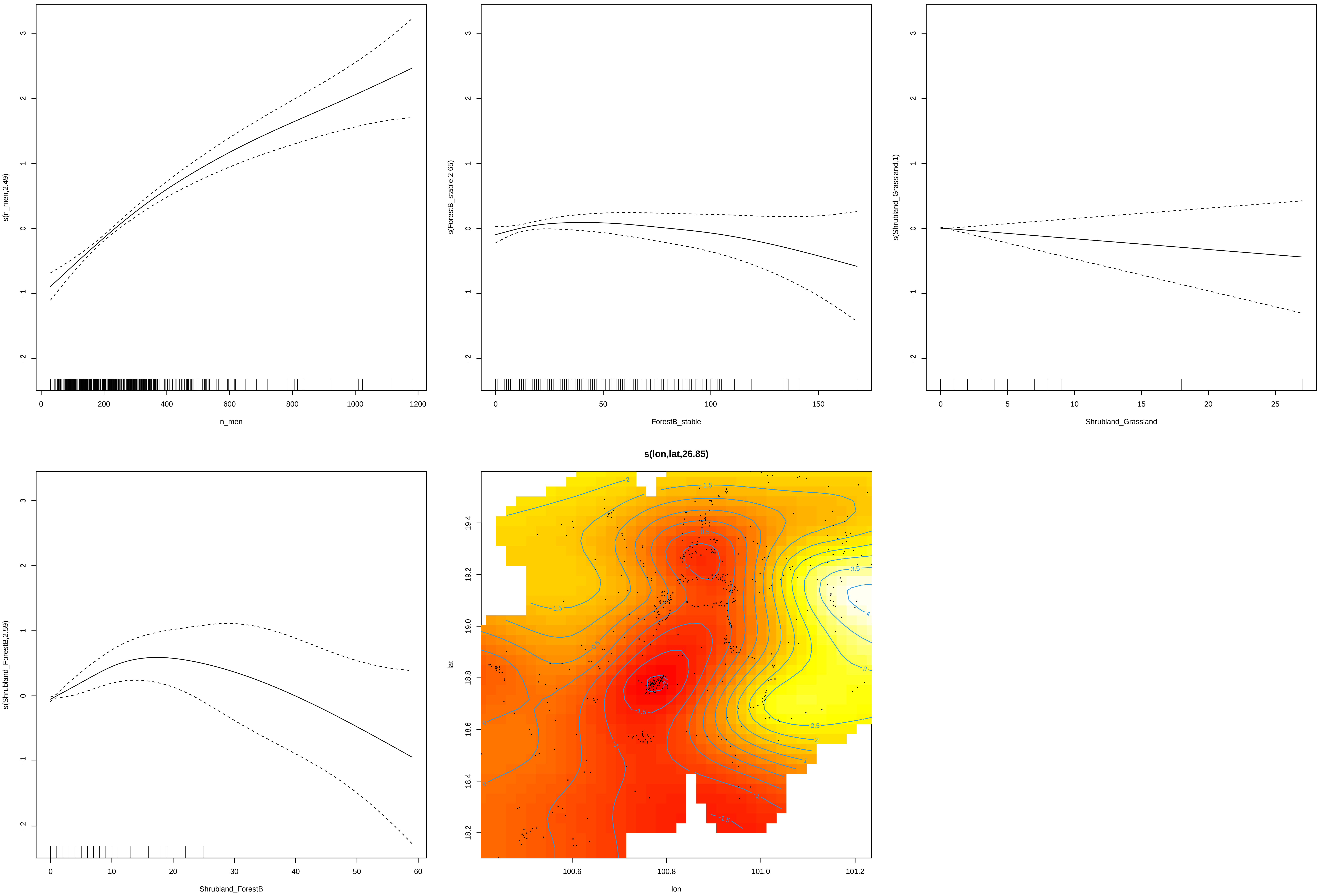

s(n_men,2.49)
n_men
0
200
400
600
800
1000
1200
3
2
1
0
−1
−2
s(ForestB_stable,2.65)
ForestB_stable
0
50
100
150
s(Shrubland_Grassland,1)
Shrubland_Grassland
0
5
10
15
20
25
s(Shrubland_ForestB,2.59)
Shrubland_ForestB
0
10
20
30
40
50
60
s(lon,lat,26.85)
lat
lon
19.4
19.2
19.0
18.8
18.6
18.4
18.2
100.6
100.8
101.0
101.2
2
1.5
3.5
4
3
2.5
-1.5

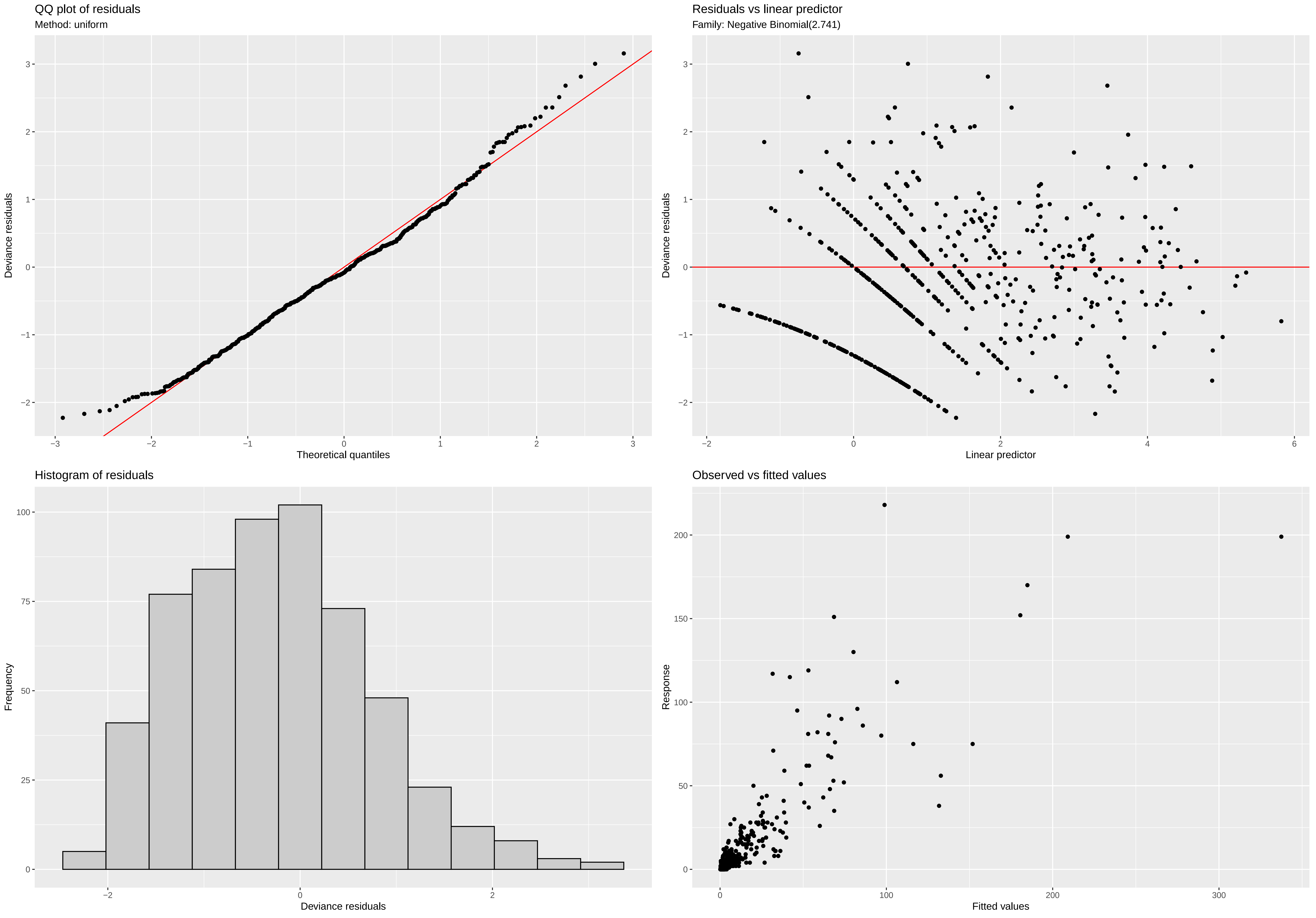
QQ plot of residuals
Method: uniform
Deviance residuals
Theoretical quantiles
−3
−2
−1
0
1
2
3
Residuals vs linear predictor
Family: Negative Binomial(2.741)
Deviance residuals
Linear predictor
−2
0
2
4
6
Histogram of residuals
Frequency
Deviance residuals
0
25
50
75
100
Observed vs fitted values
Response
Fitted values
0
50
100
150
200
300
